# Supplementary material for: Liquid metal-based synthesis of high performance monolayer SnS piezoelectric nanogenerators
Source: Nat Commun. 2020 Jul 10;11:3449. doi: 10.1038/s41467-020-17296-0 (PMC7351749; doi:10.1038/s41467-020-17296-0)
Supplement: Supplementary file 1 — Supplementary Information [file 41467_2020_17296_MOESM1_ESM.pdf]

Supplementary Information

Liquid metal-based synthesis of high performance monolayer SnS piezoelectric nanogenerators

Khan et al.

Supplementary Figures

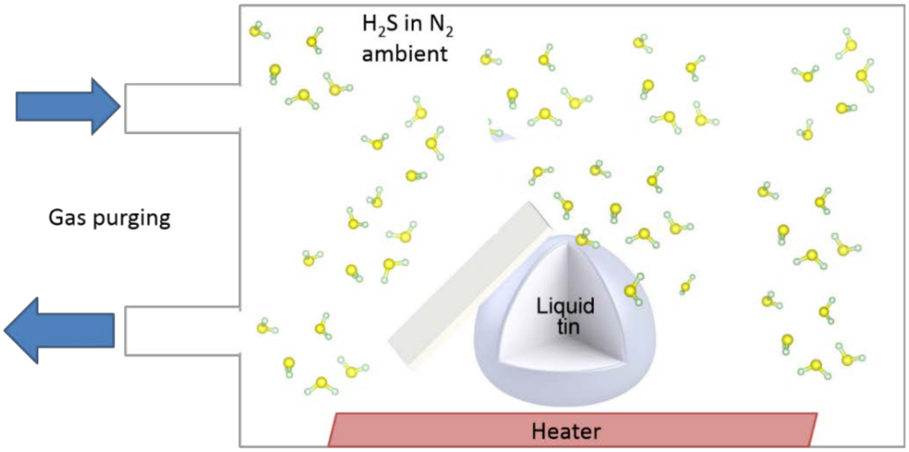

Supplementary Figure 1. The schematic of the custom-made set-up to provide an ambient S-saturated environment (using H_2S in ambient N_2) for the sulphide layer formation and its delamination.

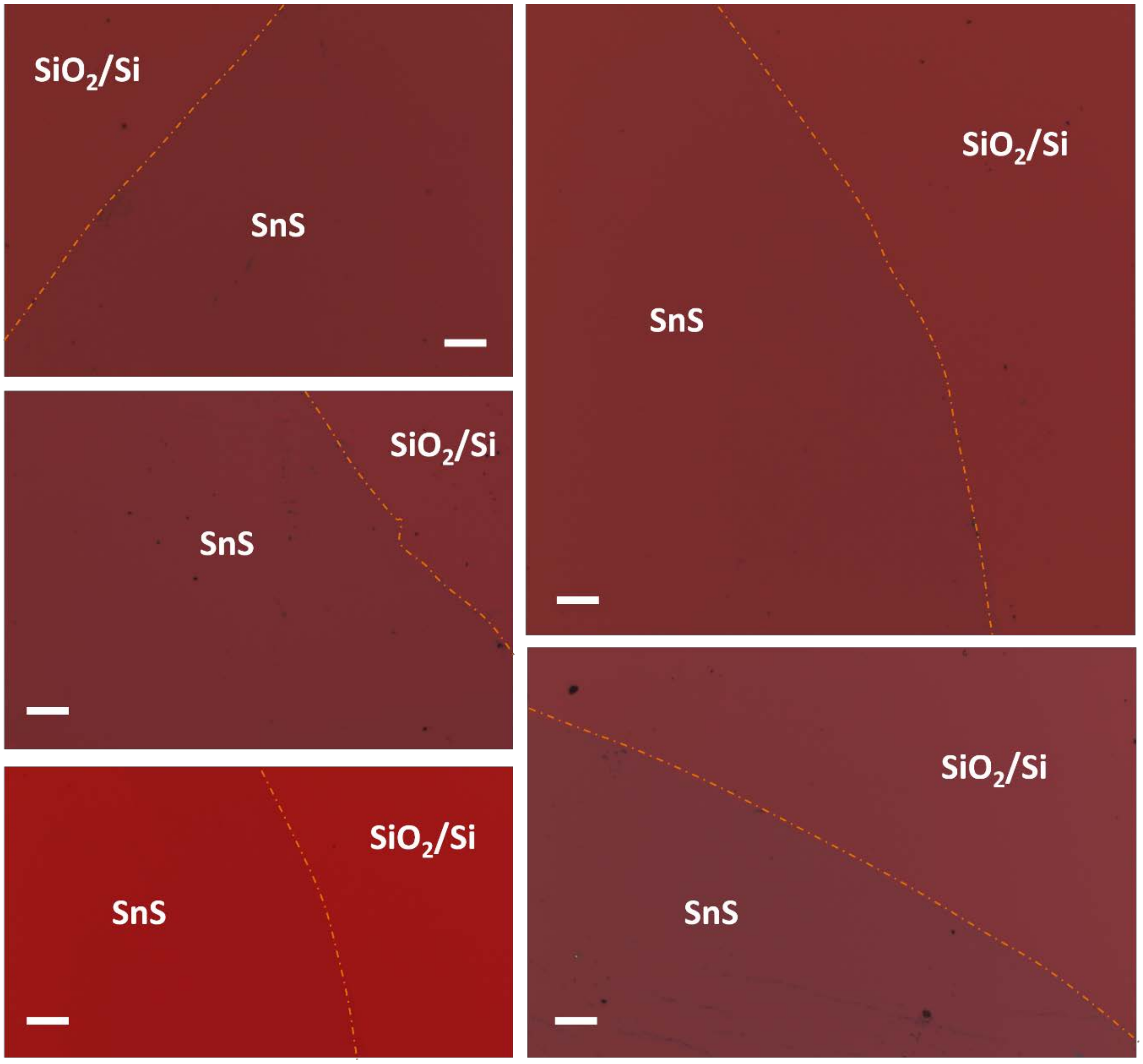

Supplementary Figure 2. Optical images of the monolayer SnS synthesised by the delamination of sulphide layer showing no cracks and consistent colour across the films, evidencing that the films thickness do not change and remain monolayer in micron size dimensions. Scale bars are 10 µm.

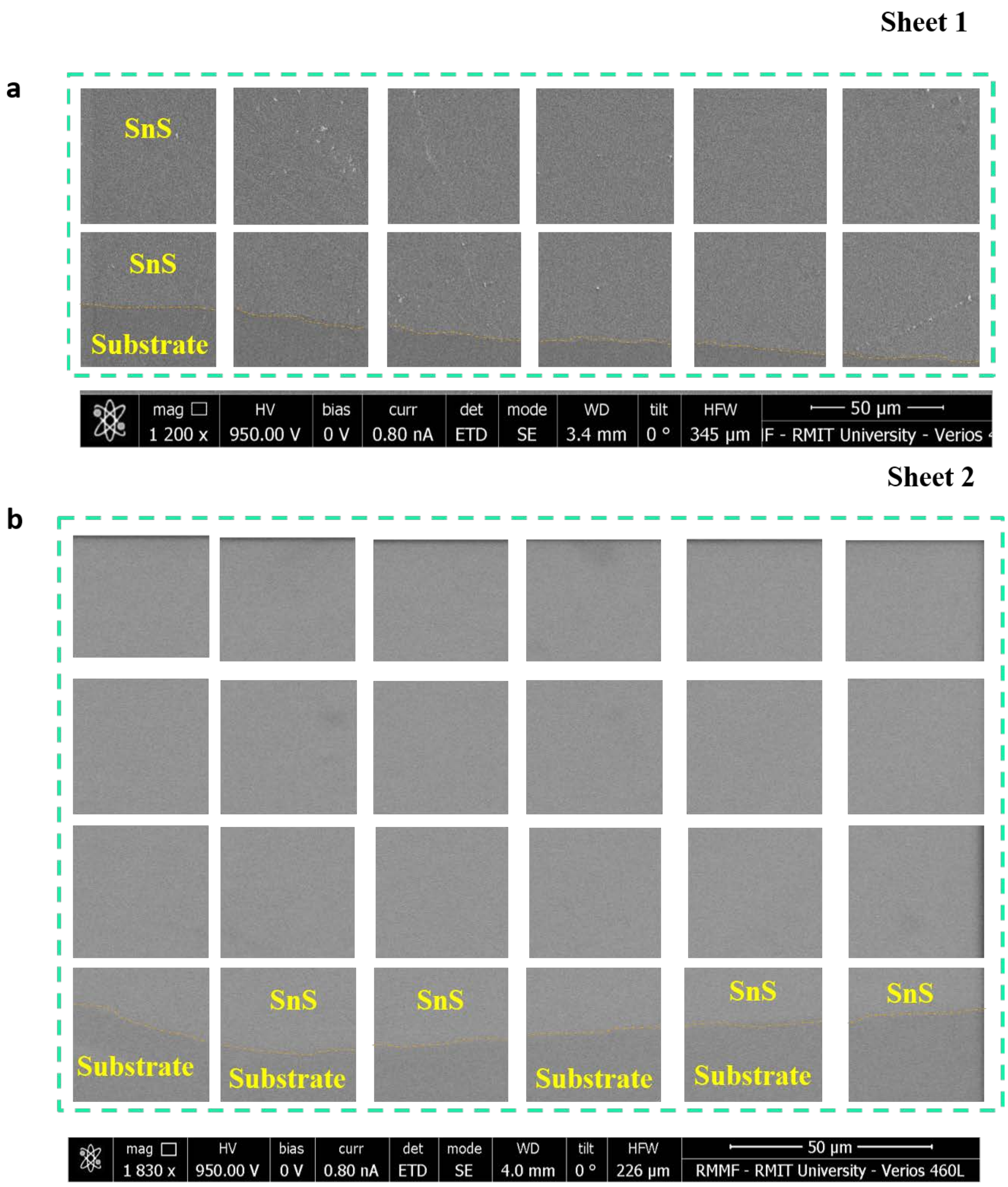

Supplementary Figure 3. Array of FESEM images of the SnS monolayer, indicating the homogeneity and high quality of the sheets presented as two examples of: a) Sheet 1 b) Sheet 2. Each area is 50 µm × 50 µm.

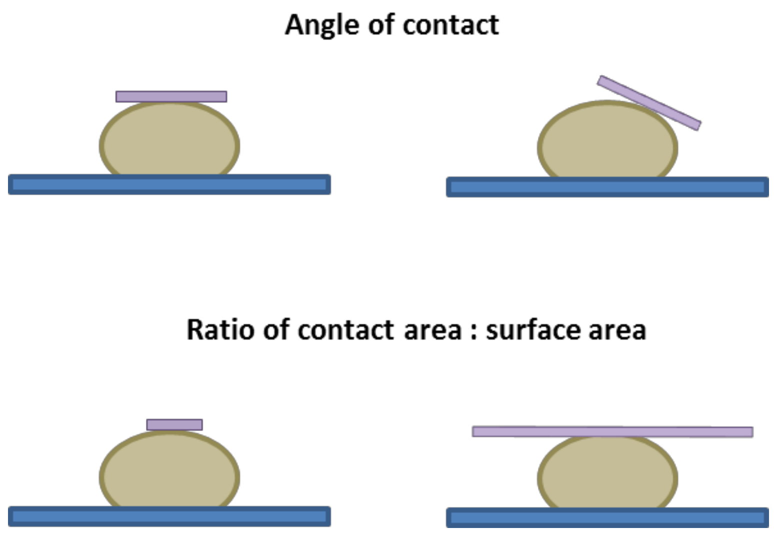

Supplementary Figure 4. Schematic illustration of factors affecting percentage transfer of SnS sheet to the substrate.

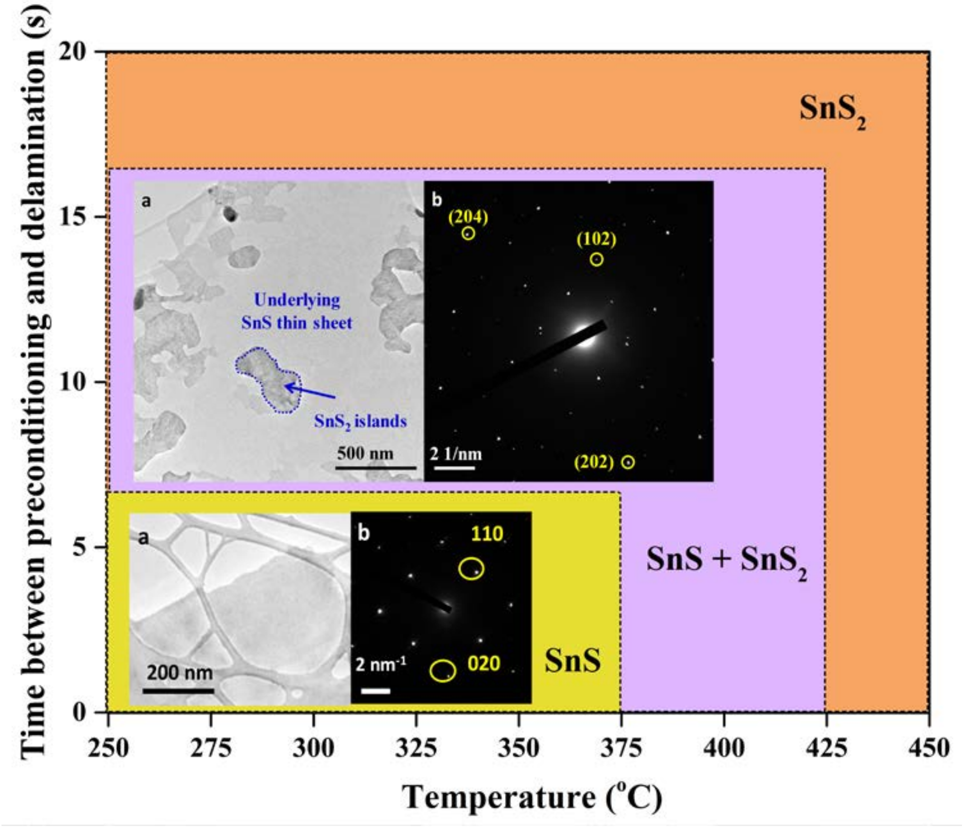

Supplementary Figure 5. Growth map illustrating the link between growth conditions and the products. Inset into each region is TEM image and SAED pattern, respectively. The SnS_2 layer can be indexed to hexagonal Berndtite-4H SnS_2 (PDF card no: 21-1231).

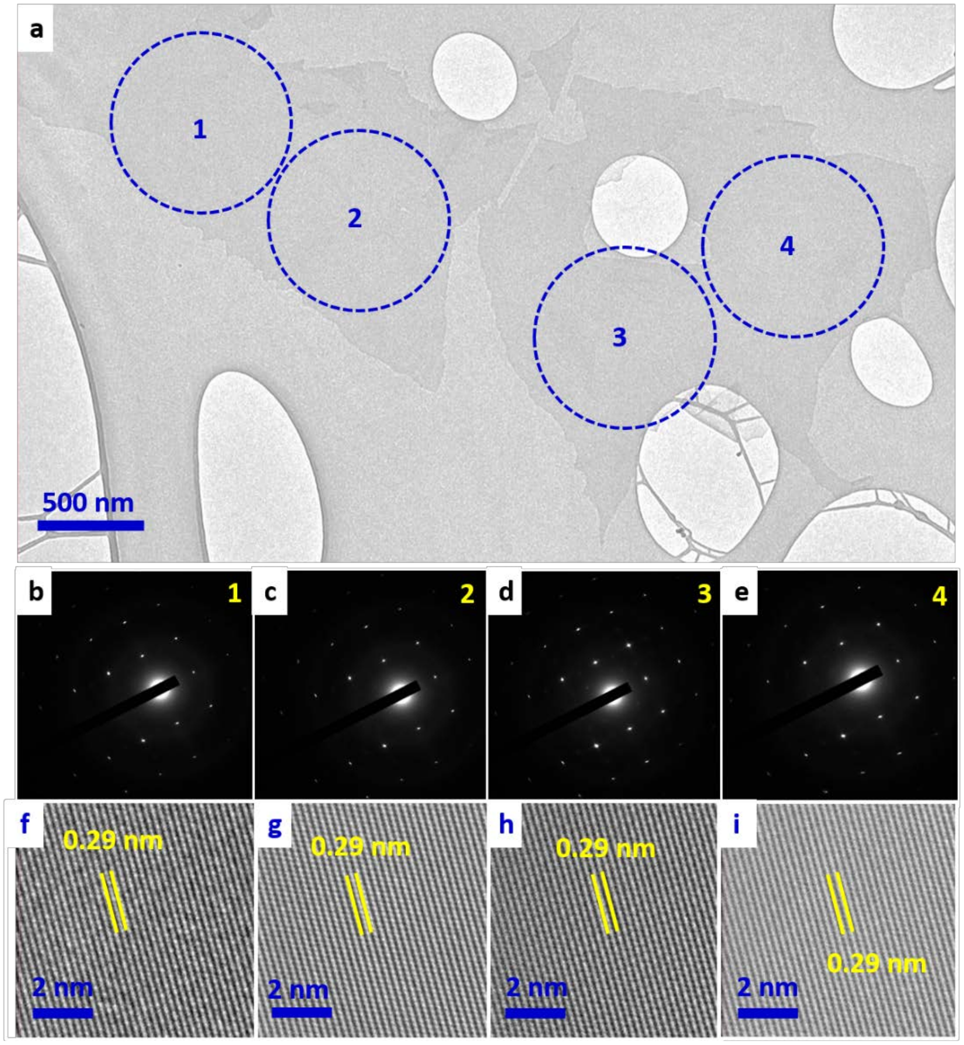

Supplementary Figure 6. a) TEM image of a large area SnS nanosheet transferred onto a TEM grid. b)–e) Four typical SAED patterns collected from the areas labelled 1–4 in a presenting closely matched lattice orientations over the whole sheet while f)-i) shows the corresponding lattice fringe spacings when zoomed into the indicated areas on the sheet.

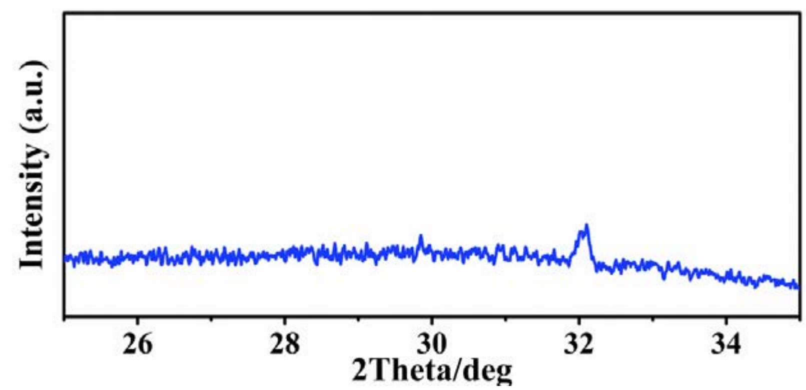

Supplementary Figure 7. XRD pattern of the one-step synthesised SnS PDF card No. 39-0354.

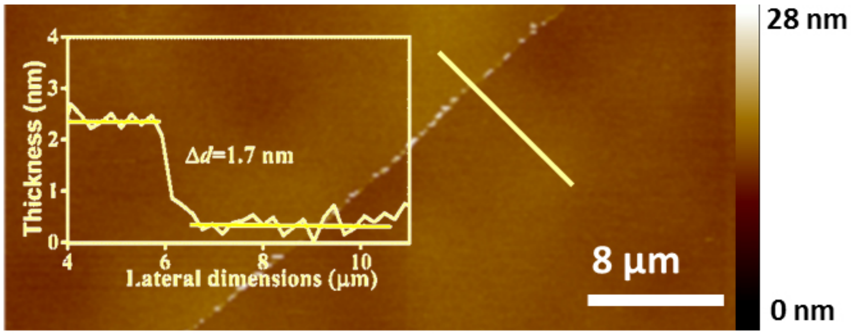

Supplementary Figure 8. AFM image of bilayer of SnS nanosheet, showing a thickness profile inset of 1.7 nm.

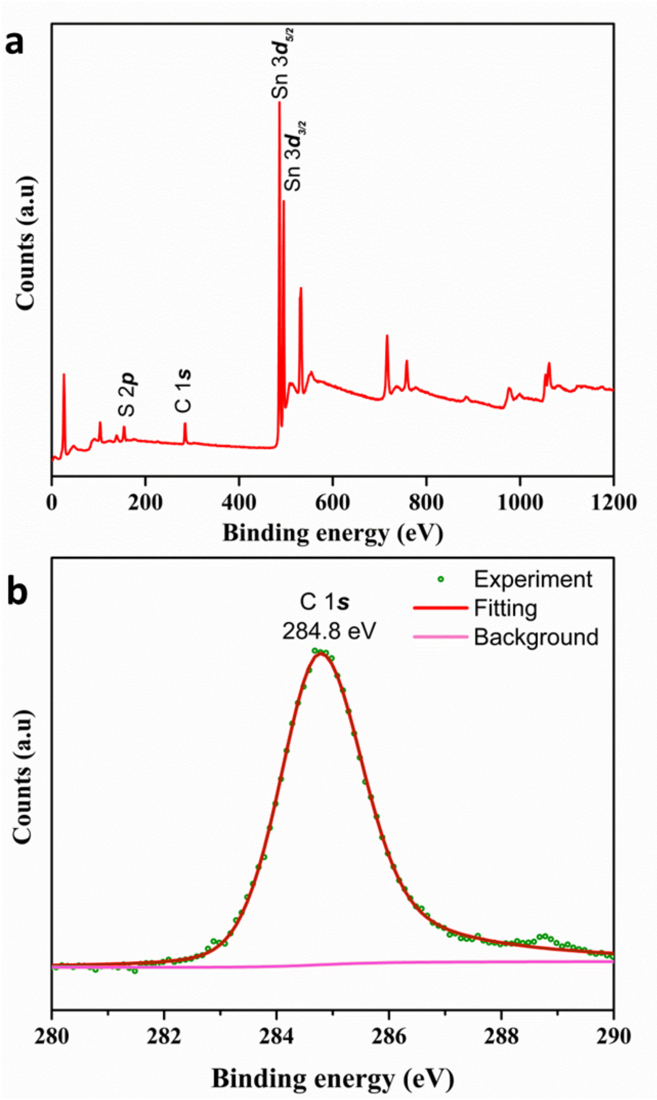

Supplementary Figure 9. XPS of SnS: a) survey spectra indicating the presence of S 2***p***, Sn 3 $\boldsymbol{d}_{5/2}$, Sn 3$\boldsymbol{d}_{3/2}$, energy states at 225.25 eV, 161.2 eV, 162.3 eV, 485.9 eV, and 494.3 eV, respectively b) C 1***s*** spectra used for the calibration of the data.

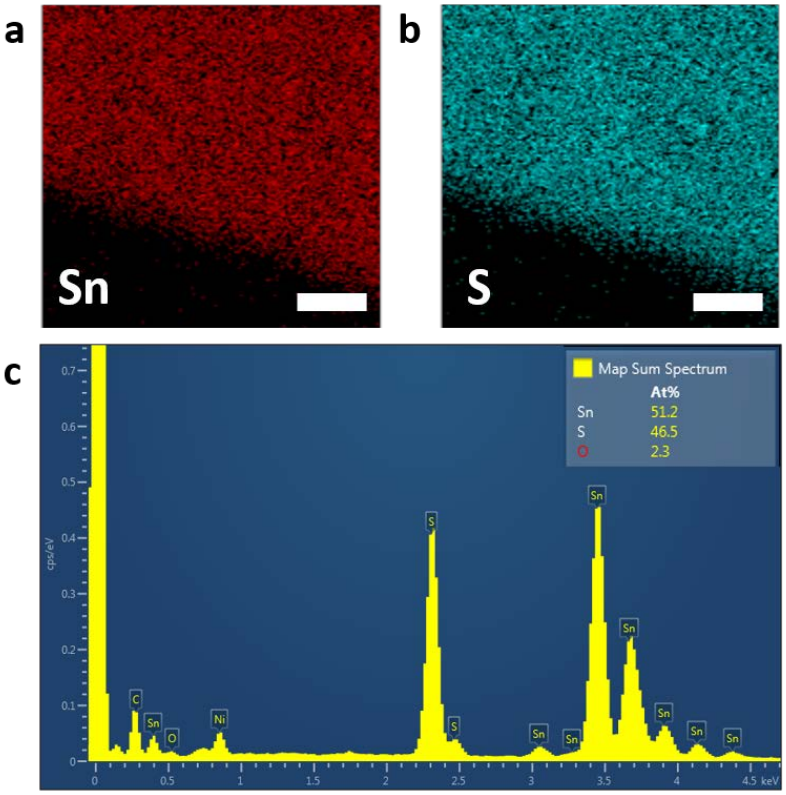

Supplementary Figure 10. EDS of monolayer SnS. Elemental mapping of a) Sn b) S with c) spectrum that shows near 1:1 ratio by spectrum. Scale bars are 100 nm.

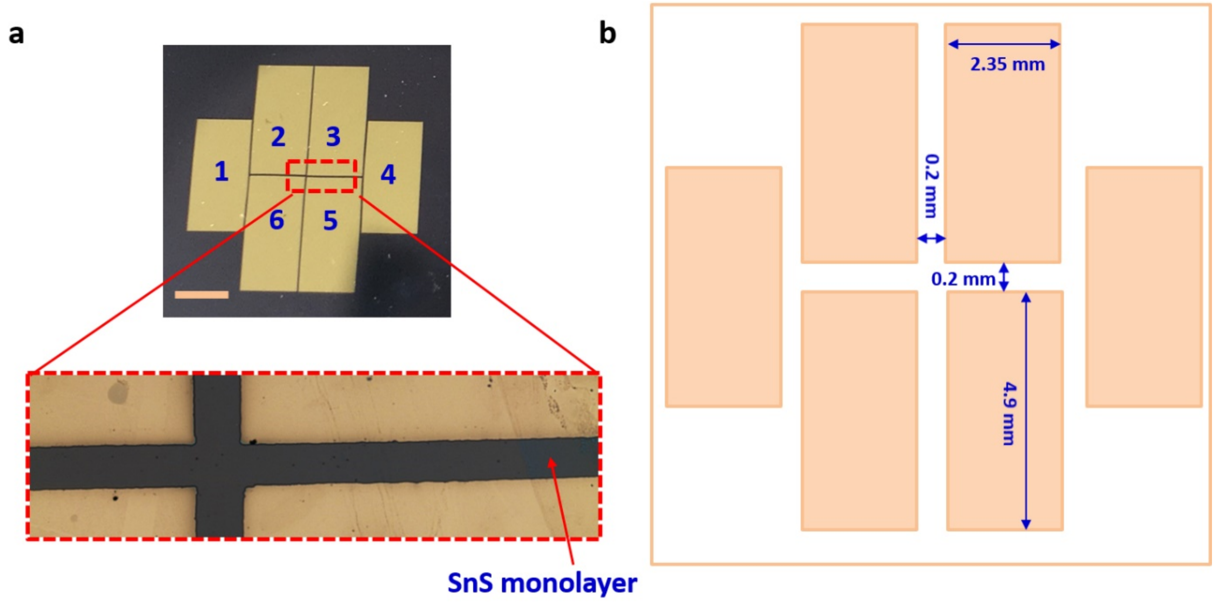

Supplementary Figure 11. Hall-effect mobility measurement: a) Optical image of the device with scale bar of 2 mm and zoom in of electrodes with SnS monolayer and b) electrode configuration with dimensions used. The image of the actual electrodes were taken at an angle and as such look slanted, which are generated from the schematic CAD file.

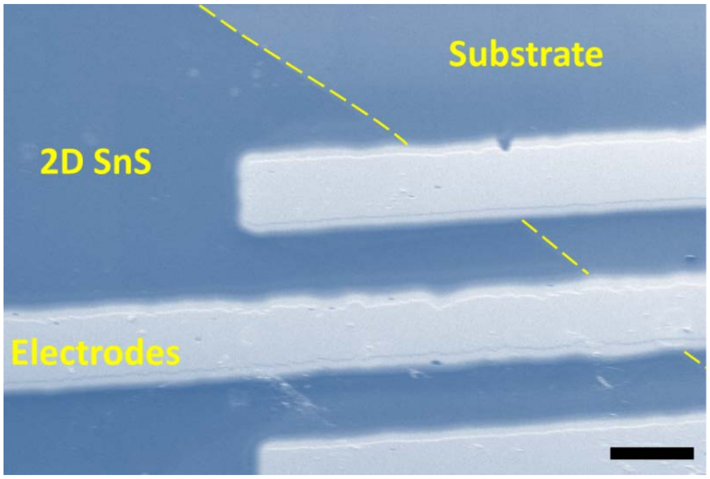

Supplementary Figure 12. SEM image of device and SnS monolayer (scale bar: 0.25 mm).

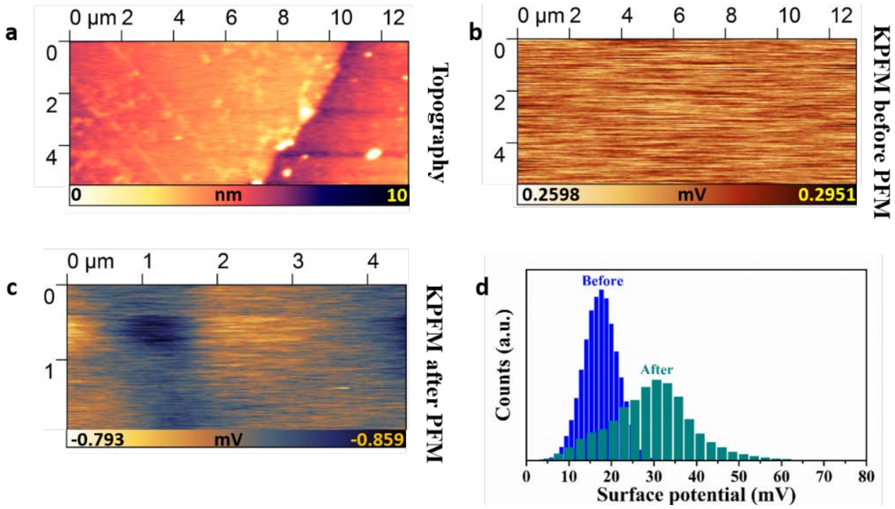

Supplementary Figure 13. Kelvin probe force microscopy (KPFM) measurements for determination of surface potential a) Topography of SnS monolayer b) KPFM measurement before the Piezoresponse force microscopy (PFM) measurement c) KPFM measurement after the PFM measurement d) Surface potential histograms indicating negligible surface potential of 15 mV before and 30 mV after the contact mode PFM analysis. There is a negligible surface potential of 15 mV initially as measured. After the PFM measurements, there is negligible residual charge build-up, which is not particularly over the flake itself and is now 30 mV.

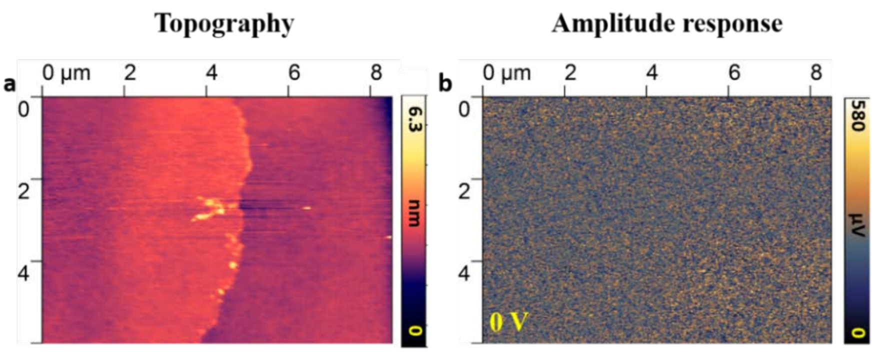

Supplementary Figure 14. PFM measurements a) Topography of the SnS monolayer b) Amplitude response when applying 0 V driving voltage depicting no response at 0 V driving amplitude. These measurements are another indication that surface charge is minimal and not of a major concern in these measurements. Another consistent precaution in all measurements performed for the PFM was the loading of the sample on a silver chuck with silver paste. A connection line of silver paste is also drawn from the flake to the chuck to ensure the sample is grounded and thus surface charge is minimal.

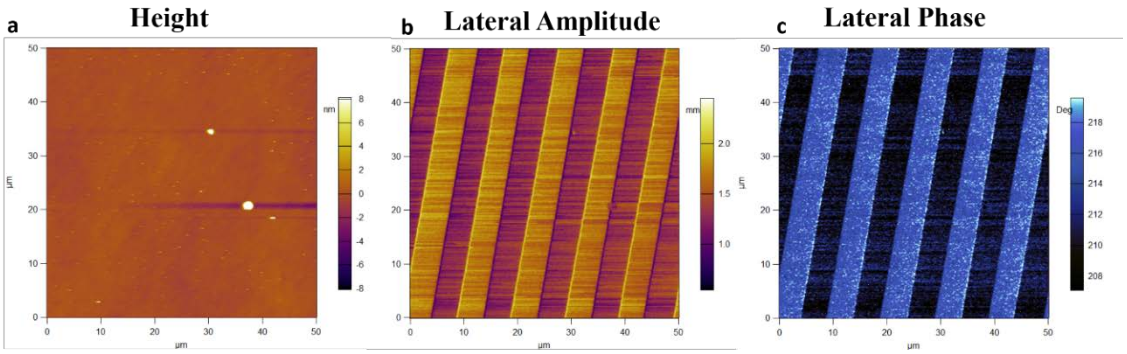

Supplementary Figure 15. PPLN sample used for calibration a) Height/ topography image b) PFM lateral amplitude c) PFM lateral phase.

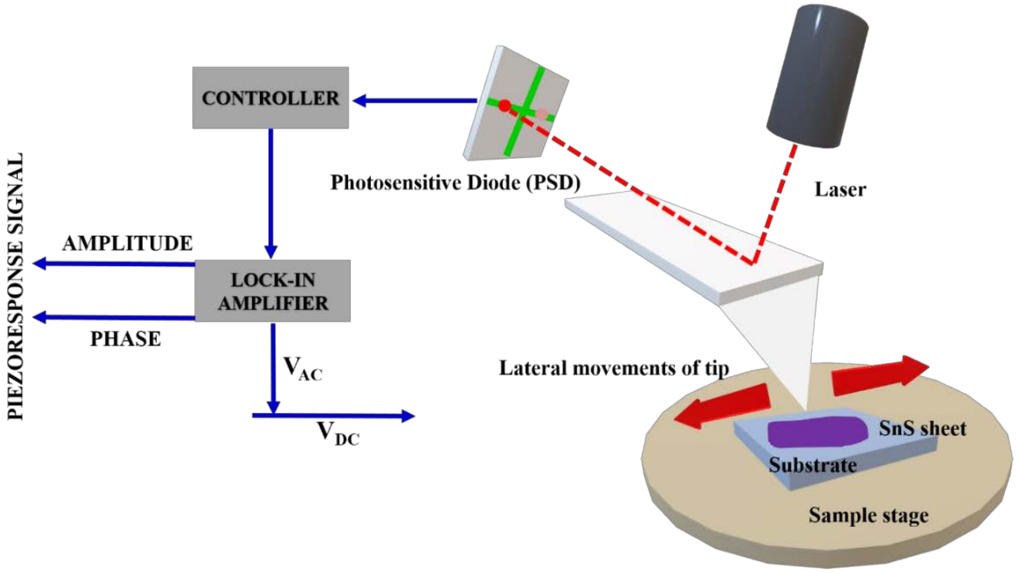

Supplementary Figure 16. PFM measurement mechanism for the lateral mode.

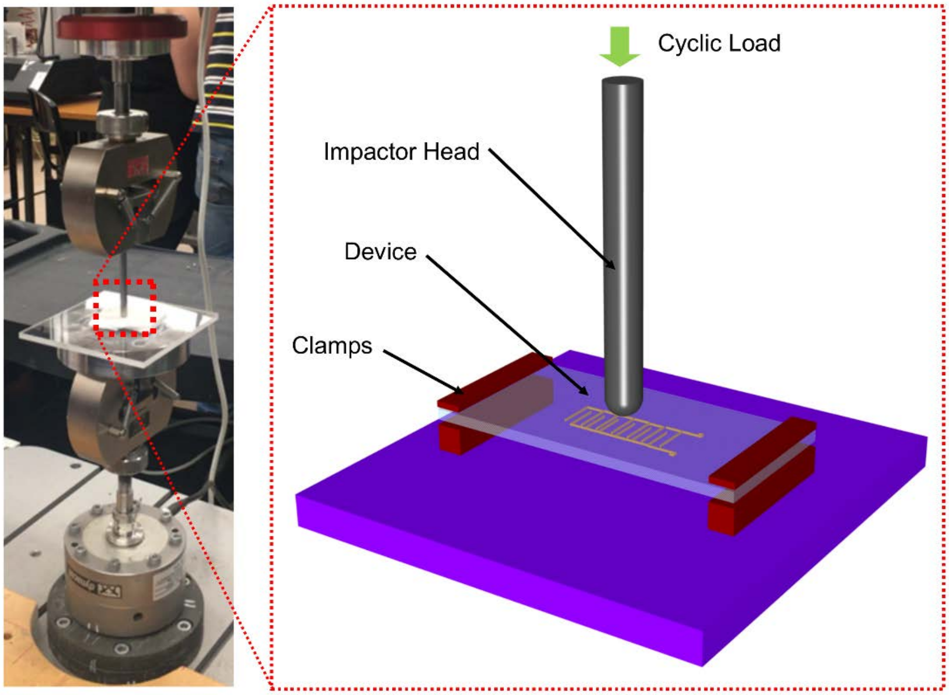

Supplementary Figure 17. Mechanical testing setup and schematic for the tapping mode.

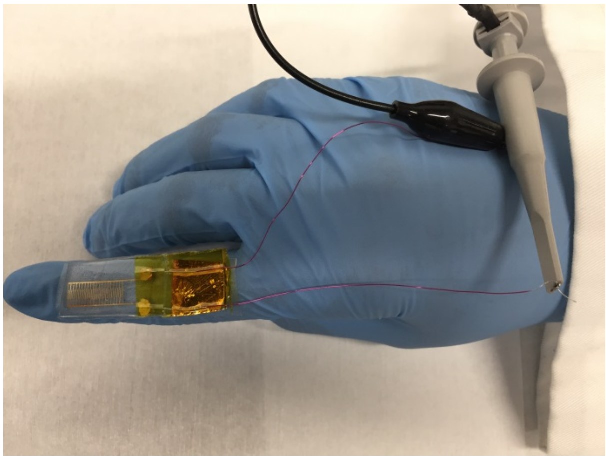

Supplementary Figure 18. Top view of the piezoelectric nanogenerator (PENG) device on hand for flexible wearable application.

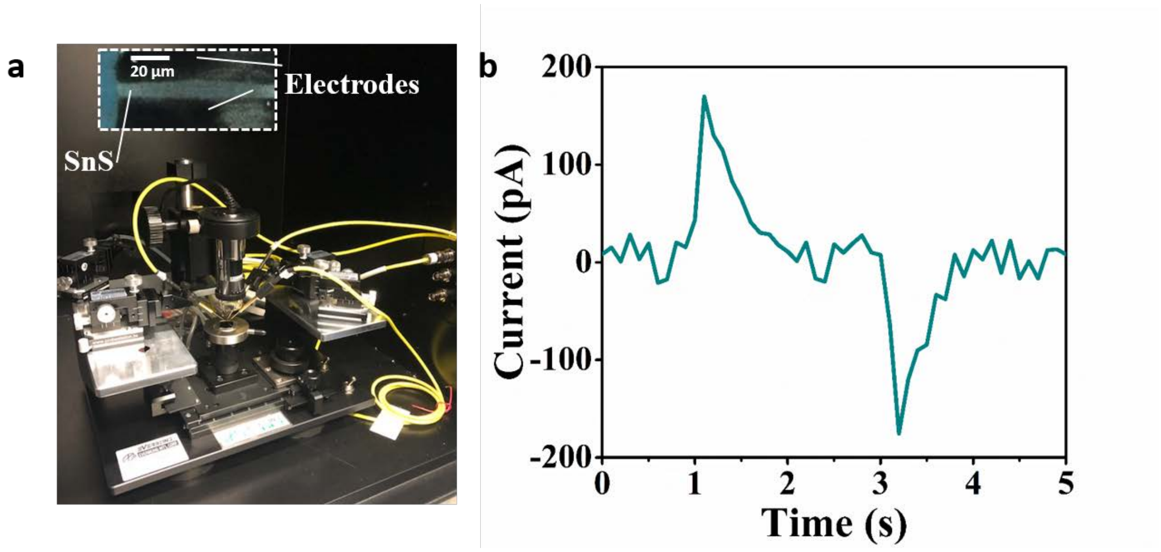

Supplementary Figure 19. Current output of the SnS monolayer device: a) Test setup for current measurement with inset of the device optical image b) Current output waveform of the device tested with 1 GΩ resistance when a force was applied at t=1 s and released at t=3 s.

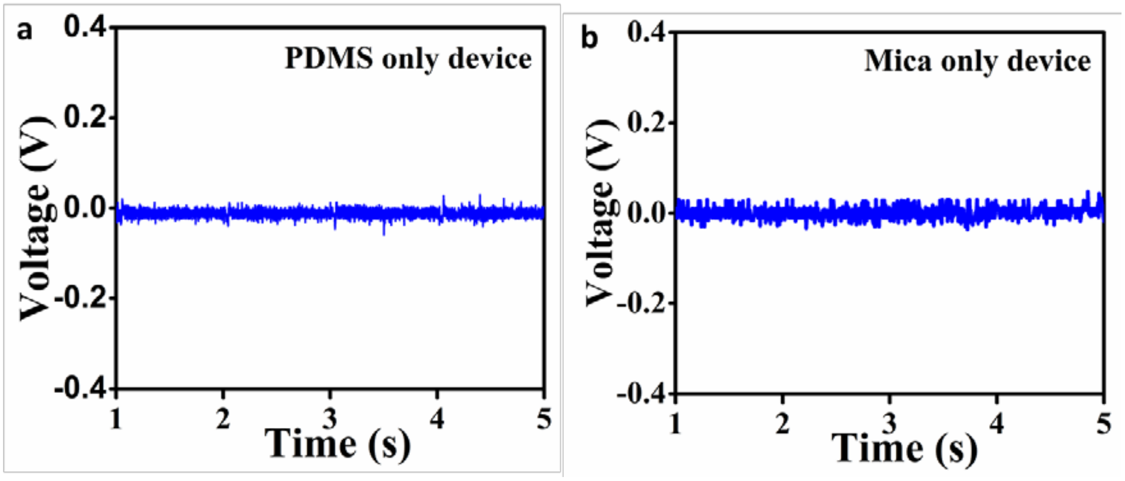

Supplementary Figure 20. Electrical outputs from bare (no SnS layer): a) PDMS device b) mica device with a force of 4 N peak-to-peak applied.

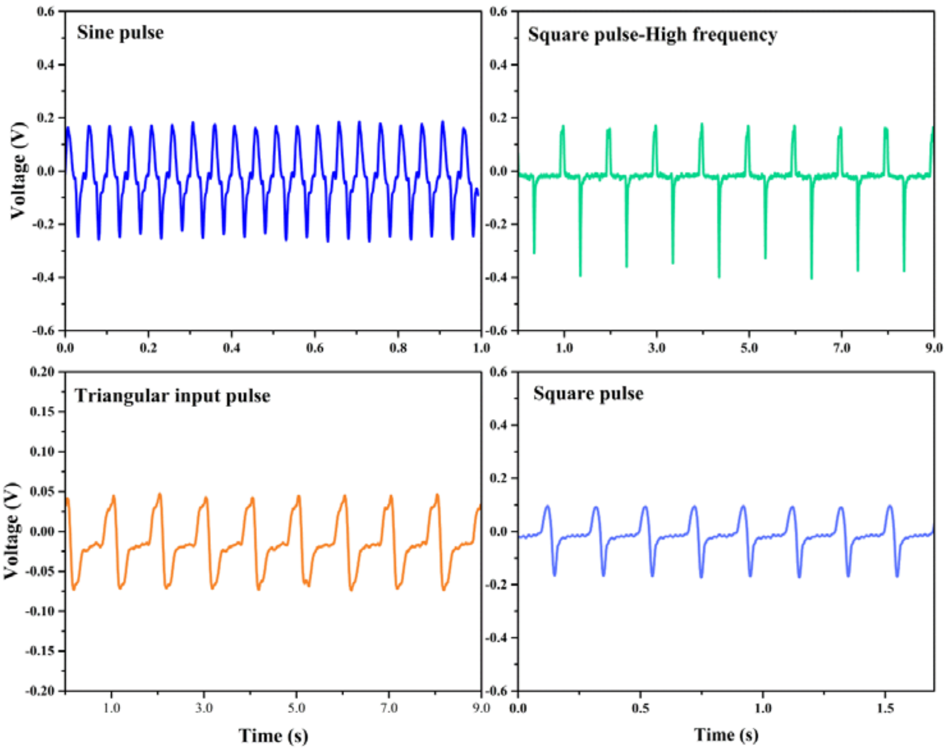

Supplementary Figure 21. Electrical voltage outputs from a range of input signal patterns.

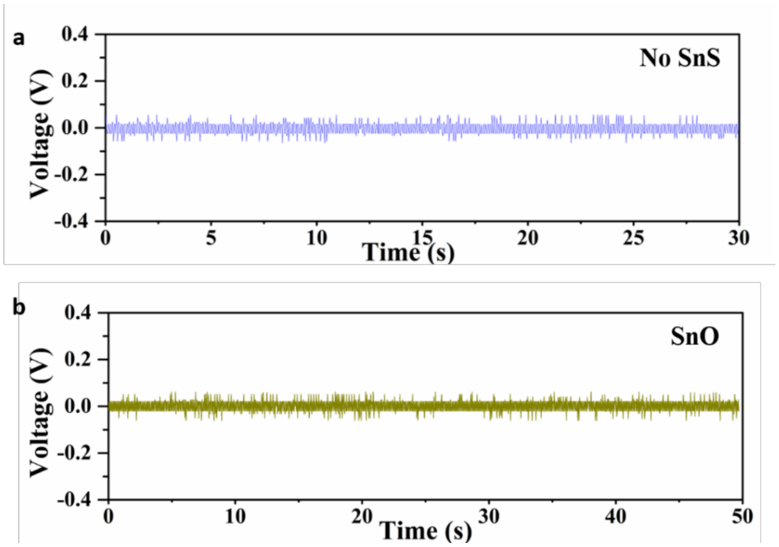

Supplementary Figure 22. Signal measured after applying mechanical stimuli with a) no monolayer SnS in between the two electrodes and b) when SnO was placed between the two electrodes.

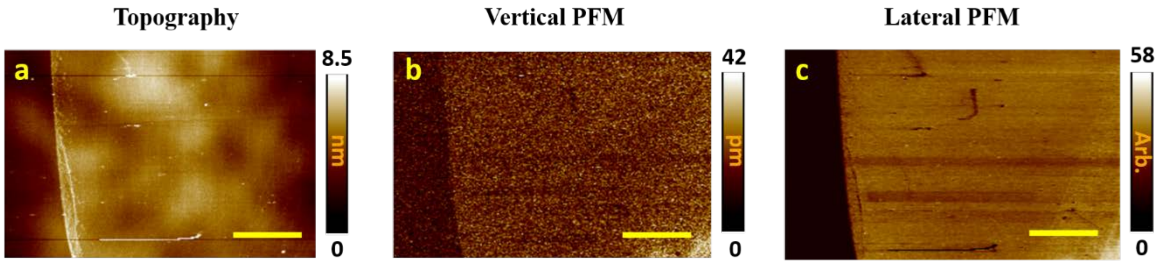

Supplementary Figure 23. PFM measurement of SnS showing a) negligible out-of-plane response in vertical PFM mode and comparatively higher in-plane response in b) Lateral PFM mode. This is expected due to the structure of the crystal. Any in-plane effect is exhibited due to piezoelectric effect whereas the negligible out-of-plane effect might be attributed to flexoelectricity [1] but this phenomenon is not dominant in monolayer structures due to the diminished strain gradient and hence not considered a dominant contributor in the device measurements. [2] Also, any such effect is more prominent in free-standing measurements. Scale bars are 8 µm.

Supplementary Notes

Supplementary Note 1. Factors affecting percentage transfer of SnS sheet to the substrate

In the exfoliation/transfer process, the actual % transfer depends on the angle of contact of the substrate with reference to the liquid metal droplet surface and also the substrate to droplet size ratio (surface area of contact region) (**Supplementary Figure 4**) . These parameters taken into account, as well as the force with which the substrate makes contact is the basis for the optimisation of the synthesis process. Our optimisation aimed towards the synthesis of large area, homogenous monolayer sheets. Cracks could arise in the use of excessive force to contact the surface, while wrinkles were mostly limited to the edges of the sheet due to the folding of the sheet during the transfer. Following such parameters the transferred area consisted of clean, homogenous monolayer sheets of SnS and the measurements were made focused on these areas. The synthesis method was further optimised to ensure no formation of byproducts by controlling the temperature and transfer time and hence these did not affect the process.

Supplementary Note 2. Discussion on the growth map illustrating the link between growth conditions and products of liquid metal based-sulphide synthesis.

Several factors are involved in the stoichiometry of the obtained tin sulphide films: temperature, and the time between the preconditioning step (when we remove a surface layer to obtain a clean surface) and film delamination and placement on a substrate. This time period is termed as ‘the transfer time’ and is measured in seconds. After the film is placed on a desired substrate, the reaction stops as there would be no precursor tin in liquid metal form available. At the critical temperature of 375ºC, and for the H_2S concentration conditions presented in the Methods section, the grown material on the surface of the liquid metal droplet is initially a thin SnS monolayer. It forms after the self-limiting reaction between the surface of liquid metal Sn and the sulfur-rich gaseous environment. At 375ºC, this monolayer SnS can be harvested at under ~7 seconds. If this formed layer is not removed, SnS_2 islands will be gradually

established on top of the SnS thin film due to the continued exposure to H_2S. If this surface reaction progresses further, eventually the islands will meet up and form a top layer of SnS_2. However, at >17 seconds SnS_2 becomes very thick and is the dominating material within the film and no SnS can be practically detected. The second factor is the temperature at which the droplet is heated. At 250ºC to 375ºC, monolayer SnS, a combination of SnS and SnS_2 islands and then eventually SnS_2 films are obtained depending on the waited time. However, when it is above 375ºC, the conditions are conducive for a faster reaction with the sulfur source in the ambient environment and hence the material grown on the surface of the droplet is SnS_2. A growth map is illustrated in **Supplementary Figure 5**. highlighting the important time and temperature parameters for the synthesis process. It is important to note that the layer reaction stops the moment when it is delaminated and transferred onto a substrate. The transferred film on a substrate does not change its stoichiometry or thickness. In the monolayer SnS case, it remains a stable homogenous monolayer on the surface in the normal ambient condition.

Supplementary Note 3. Further analysis of single-crystallinity, reduced grain boundaries and homogeneity of as-synthesised SnS sheets

Selected-area electron diffraction (SAED) patterns at 4 typical locations on the transferred sheet (marked 1 to 4 in **Supplementary Figure 6 a**) were generated to assess the single crystallinity of these monolayers. These areas showed the same crystallographic orientations (**Supplementary Figure 6 b-e**) and the corresponding lattice fringe patterns (**Supplementary Figure 6 f-i**) taken zoomed into these areas. These further characterisations indicate the large area homogenous single crystalline domains (at least >1 μm) and minimal grain boundaries in the sheets. The two adjacent sheets chosen for this analysis were deliberately chosen to demonstrate that the sheets from one touch of the grid result in large area single crystals even if the sheets break during the synthesis process. The transfer from the droplet to the amorphous carbon filmed grid is not as ideal as the transfer onto a SiO_2/Si substrate, hence the sheets on the TEM grid are broken and comparatively smaller than the ones obtained on rigid substrates.

Supplementary Note 4. Discussion on calculation of Sn:S ratio

We found that the ratio obtained by XPS always shows that S is less than Sn. The loss of sulphur may be due the intensity of the X-ray resulting in a depleted S ratio due to the extremely thin layer as this is also observed in self-assembling monolayers. Another factor is the overlapping of the Si2***s*** plasmon peak with the S2***p*** spectra. Mostly, interpreting the S 2***p*** spectrum on silicon-based substrates is challenging due to the satellite peaks induced from the proximal Si 2***s*** (~152 eV), which is a result of surface plasmon (~17 eV) being excited by the photoelectrons [3-6]. Hence, obtaining a clean S 2***p*** peak is often difficult on silicon surfaces which distorts ratio analysis to some extent. The lower S content as well as the correct binding values of the Sn for the 2+ valency state confirms that the synthesised material is not SnS_2 which is also confirmed by Raman spectroscopy. We have further conducted EDS mapping to calculate the ratio instead and it shows an approximately 1:1 ratio (**Supplementary Figure 10**).

Supplementary Tables

Supplementary Table 1. Comparison synthesis methods used for synthesising pristine 2D Group IV monochalcogenides

Synthesis method	Material	Shortcoming	Refs
Micromechanical cleavage/tape exfoliation	SnS	Cannot achieve less than 10 layers thickness	7,8
Crystal synthesis	SnS	Cannot achieve less than 10 layers thickness	9-14
Liquid-phase exfoliation	SnS, SnSe	Prolonged ultrasonication resulting small lateral dimensions	15-18
Au-mediated exfoliation with/or application of post-thinning techniques such as laser or thermal annealing	SnS, SnSe, GeSe	SnS flakes less than ~4 nm were completely oxidised. Forms SnO_x/SnS (SnO-passivated heterostructure) which was unsuitable to measure a correct output nanogenerator response	13,19,20

Supplementary Table 2. Table of important parameters of previous reports on 2D material-based nanogenerators

2D Material	Parameters					Ref	Piezoelectric coefficients	
	**V**	**I**	**Maximum instantaneous power @ stated load resistance**	**Test conditions**	**Power density**			
	**Units**						**Theoretical**	**Experimental**
	**mV**	**pA**	**pW**	**% strain**	**mWm^{-2}**		**pmV^{-1}**	
Monolayer -MoS_2	18 (peak OC voltage)	27 (peak SC current)		0.64		21	d_{11}=3.65 [22]<br>d_{11}=4.94 [23]<br>d_{11}=3.73 [24]	
	3.5 (calc)	12.5 (calc)	55.3 fW (220 MΩ)	0.53	2			
Monolayer -MoS_2 (armchair direction)	20 (peak output)	30 (peak output)		0.48, 0.5 Hz, 70mm		25	d_{11}=3.65 [22]<br>d_{11}=4.94 [23]<br>d_{11}=3.73 [24]	d_{11}=3.78 (AC)[25]
Monolayer -MoS_2 (zigzag direction)	10 (peak output)	20 (peak output)						d_{11}=1.38 (ZZ)[25]

Monolayer -MoS_2 (armchair direction)	20 (1 GΩ) graph		0.4 (1 GΩ)-graph		0.5			
Monolayer MoS_2 (zigzag direction)	10 mV (1 GΩ) graph		0.2 (1 GΩ)-graph		0.25			
Monolayer -WSe_2	~35 (calc)	72 (calc)	2.54 (500 MΩ)	0.39	0.5		d_{11}=2.79	d_{11}=3.26 ± 0.3 [26]
Turbostratic-WSe_2	~57 (graph)	71 (calc)	4.05 (~ 0.1 GΩ)	0.89	0.8	26		d_{11} =0-1.5 [26]
Monolayer - WSe_2	~37 (graph)	55 (calc)	2.05 (~ 0.5 GΩ)	0.89	0.4			
Pristine MoS_2	10 (1GΩ)	30 (output) 1GΩ	0.07 (500 MΩ)	0.48	0.007	27	d_{11}=3.65 [22] d_{11}=4.94 [23] d_{11}=3.73 [24]	d_{11}=3.06 ± 0.6 [27]
S-treated MoS_2	20 (1GΩ)	100 (output) 1GΩ	0.73 (500 MΩ)	0.48	0.073			d_{11}=3.73 ± 0.2[27]
Monolayer -SnS	~150 (1GΩ)	160 (output) (1GΩ)	24 (1 GΩ)	0.70	24	This work	d_{11}=144.76[28] d_{12}= -22[28]	PFM measured value: 26.1 ± 0.3

Note: Values in green were calculated or interpreted by the authors. AC- armchair, ZZ- zigzag

Supplementary References

1 Wang, X. *et al.* Probing effective out-of-plane piezoelectricity in van der Waals layered materials induced by flexoelectricity. *Small* **0**, 1903106.

2 Zubko, P., Catalan, G. & Tagantsev, A. K. Flexoelectric effect in solids. *Annu. Rev. Mater. Res.* **43**, 387-421 (2013).

3 Zhang, Y. F. *et al.* Electronic structure of silicon nanowires: A photoemission and x-ray absorption study. *Phys. Rev. B* **61**, 8298-8305 (2000).

4 Mannella, N., Gabetta, G. & Parmigiani, F. Plasmon energy shift in porous silicon measured by x-ray photoelectron spectroscopy. *Appl. Phys. Lett.* **79**, 4432-4434 (2001).

5 Kamineni, H. S. *et al.* Optical and structural characterization of thermal oxidation effects of erbium thin films deposited by electron beam on silicon. *J. Appl. Phys.* **111**, 013104 (2012).

6 Lee, C.-H., Chen, W.-C. & Khung, Y. XPS analysis of 2-and 3-aminothiophenol grafted on silicon (111) hydride surfaces. *Molecules* **23**, 2712 (2018).

7 Sucharitakul, S. *et al.* Screening limited switching performance of multilayer 2D semiconductor FETs: the case for SnS. *Nanoscale* **8**, 19050-19057 (2016).

8 Higashitarumizu, N., Kawamoto, H., Ueno, K. & Nagashio, K. Fabrication and surface engineering of two-dimensional SnS toward piezoelectric nanogenerator application. *MRS Adv.* **3**, 2809-2814 (2018).

9 Xia, J. *et al.* Physical vapor deposition synthesis of two-dimensional orthorhombic SnS flakes with strong angle/temperature-dependent Raman responses. *Nanoscale* **8**, 2063-2070 (2016).

10 Tian, Z., Guo, C., Zhao, M., Li, R. & Xue, J. Two-dimensional SnS: A phosphorene analogue with strong in-plane electronic anisotropy. *ACS Nano* **11**, 2219-2226 (2017).

11 Li, M. *et al.* Revealing anisotropy and thickness dependence of Raman spectra for SnS flakes. *RSC Adv.* **7**, 48759-48765 (2017).

12 Pei, T. *et al.* Epitaxy of ultrathin SnSe single crystals on polydimethylsiloxane: in-plane electrical anisotropy and gate-tunable thermopower. *Adv. Electron. Mater.* **2**, 1600292 (2016).

13 Jizhou, J. *et al.* Two-step fabrication of single-layer rectangular SnSe flakes. *2D Mater.* **4**, 021026 (2017).

14 Cheng, C.-H. *et al.* Catalytically solid-phase self-organization of nanoporous SnS with optical depolarizability. *Nanoscale* **8**, 4579-4587 (2016).

15 Ye, Y. *et al.* Linear and nonlinear optical properties of few-layer exfoliated SnSe nanosheets. *Adv. Opt. Mater.* **7**, 1800579 (2019).

16 Brent, J. R. *et al.* Tin(II) sulfide (SnS) nanosheets by liquid-phase exfoliation of herzenbergite: IV–VI main group two-dimensional atomic crystals. *J. Am. Chem. Soc.* **137**, 12689-12696 (2015).

17 Sun, Y. *et al.* All-surface-atomic-metal chalcogenide sheets for high-efficiency visible-light photoelectrochemical water splitting. *Adv. Energy Mater.* **4**, 1300611 (2014).

18 Li, L. *et al.* Single-layer single-crystalline SnSe nanosheets. *J. Am. Chem. Soc.* **135**, 1213-1216 (2013).

19 Higashitarumizu, N. *et al.* Self-passivated ultra-thin SnS layers via mechanical exfoliation and post-oxidation. *Nanoscale* **10**, 22474-22483 (2018).

20 Zhao, H. *et al.* Band structure and photoelectric characterization of GeSe monolayers. *Adv. Funct. Mater.* **28**, 1704855 (2018).

21 Wu, W. *et al.* Piezoelectricity of single-atomic-layer MoS_2 for energy conversion and piezotronics. *Nature* **514**, 470 (2014).
22 Blonsky, M. N., Zhuang, H. L., Singh, A. K. & Hennig, R. G. Ab Initio Prediction of Piezoelectricity in Two-Dimensional Materials. *ACS Nano* **9**, 9885-9891 (2015).
23 Alyörük, M. M., Aierken, Y., Çakır, D., Peeters, F. M. & Sevik, C. Promising piezoelectric performance of single layer transition-metal dichalcogenides and dioxides. *J. Phys. Chem. C* **119**, 23231-23237 (2015).
24 Duerloo, K.-A. N., Ong, M. T. & Reed, E. J. Intrinsic piezoelectricity in two-dimensional materials. *J. Phys. Chem. Lett.* **3**, 2871-2876 (2012).
25 Kim, S. K. *et al.* Directional dependent piezoelectric effect in CVD grown monolayer MoS_2 for flexible piezoelectric nanogenerators. *Nano Energy* **22**, 483-489 (2016).
26 Lee, J.-H. *et al.* Reliable piezoelectricity in bilayer WSe_2 for piezoelectric nanogenerators. *Adv. Mater.* **29**, 1606667 (2017).
27 Han, S. A. *et al.* Point-defect-passivated MoS_2 nanosheet-based high performance piezoelectric nanogenerator. *Adv. Mater.* **30**, 1800342 (2018).
28 Fei, R., Li, W., Li, J. & Yang, L. Giant piezoelectricity of monolayer group IV monochalcogenides: SnSe, SnS, GeSe, and GeS. *Appl. Phys. Lett.* **107**, 173104 (2015).
